# Mixed reality applications in percutaneous stone surgery: A systematic review and meta‐analysis of stone‐free rate and perioperative complications

**DOI:** 10.1002/bco2.70278

**Published:** 2026-09-21

**Authors:** Christopher Yong‐Zyn Lo, Frida Toscano, John Joson Ng, Du Jingzeng, Archan Khandekhar, Hemendra Shah, Robert Marcovich, Julio Ojalvo, Sarvesh Saini, Ubbo Visser, Jonathan Katz

**Affiliations:** ^1^ Department of Urology Singapore General Hospital Singapore; ^2^ Anahuac North University Mexico City Mexico; ^3^ Duke‐NUS Medical School Singapore; ^4^ Department of Urology University of Miami Coral Gables Florida USA; ^5^ Department of Computer Science University of Miami Coral Gables Florida USA

**Keywords:** augmented reality, complications, ECIRS, mixed reality, PCNL, percutaneous stone surgery, stone‐free rate, virtual reality

## Abstract

**Objectives:**

The aim of this study is to evaluate the effect of mixed reality (MR)‐assisted percutaneous nephrolithotomy (PCNL) on stone‐free rate (SFR), perioperative complications and operative efficiency.

**Methods:**

A search of PubMed, Embase and Web of Science from inception to February 2026 followed PRISMA guidelines. Studies comparing MR‐assisted versus standard fluoroscopic or ultrasound‐guided PCNL were included. Risk of bias was assessed using RoB2 and ROBINS‐I. Random‐effects meta‐analyses calculated standardized mean differences (SMD) or risk ratios (RR) with 95% confidence intervals (CI).

**Results:**

Nine studies (*n* = 774; 378 MR‐assisted, 396 control) met inclusion criteria; eight studies (*n* = 730; 356 MR‐assisted, 374 control) were included in meta‐analysis. MR modalities included immersive virtual reality (VR) planning, holographic MR and CT‐ultrasound (US) fusion navigation. The MR‐assisted group had modest improvements in SFR compared to the control group (RR = 1.09; 95% CI 1.00–1.18; *p* = 0.044; *I* ^2^ = 17.3%, *p* = 0.29). Complication rates were similar between MR‐assisted and standard approaches. There was no statistically significant difference in puncture time between MR‐assisted and standard groups (SMD = −0.64; 95% CI −2.22 to 0.93; *p* = 0.42), with considerable heterogeneity. Similarly, there was no statistically significant difference in operative duration (SMD = −0.22; 95% CI ‐0.66 to +0.23; *p* = 0.34). While not meeting the threshold for pooled meta‐analysis, narratively, we found that MR‐assistance also reduced puncture attempts, blood loss and fluoroscopy time.

**Conclusions:**

MR‐assisted PCNL is associated with an apparent increase in SFR while maintaining comparable operative parameters and complication rates compared with standard techniques. Heterogeneity in these technologies and their applications limits generalizability. Larger, adequately powered standardized trials are recommended to interrogate key outcome measures including SFR and complication rates.

## 1 INTRODUCTION

The field of endourology has evolved rapidly since the early 1990s, when Washida first described the use of video endoscopy to visualize the renal collecting system and facilitate minimally invasive intervention for urolithiasis.^1^ Building upon these early innovations, mixed reality (MR) technologies represent a new frontier for enhancing visualization, guidance and precision in urologic surgery.

MR encompasses the continuum between the real and virtual environments that includes augmented reality (AR) and virtual reality (VR).^2^ AR refers to the ‘superimposition of preoperative or intraoperative images into the operative field’.^3^ Alternatively, VR generates an immersive, completely artificial computer‐simulated image and environment with real‐time interaction.^4^ Interest in MR applications has increased in recent years across multiple urological domains, including prostate, renal, ureteral, adrenal and penile surgeries, offering potential benefits for both surgical planning and intraoperative execution.^3^, ^5^, ^6^

Within endourology, percutaneous nephrolithotomy (PCNL) is a commonly performed treatment for large or complex stones. In both European Association of Urology (EAU) and American Urological Association (AUA) guidelines, PCNL is generally recommended as first‐line treatment for kidney stones larger than 15–20 mm.^7^ Standard antegrade PCNL is also increasingly combined with a simultaneous retrograde flexible ureteroscopic approach to improve single‐session clearance of complex stones while limiting the number of percutaneous tracts in endoscopic combined intrarenal surgery (ECIRS).

Despite its efficacy, PCNL carries risks of complications such as haemorrhage and perforation, which are more frequent in patients with multiple comorbidities or complex anatomy.^8^ Furthermore, while urologists are increasingly likely to obtain their own PCNL access, with the proportion in the United States rising from 12.8% in 2007 to 32.3% in 2017, the process remains technically challenging and is still performed by radiologists in many centres.^9^ Given these challenges, there is growing interest in leveraging MR technologies in PCNL for enhanced intraoperative guidance and puncture precision, potentially reducing complication rates.

Proposals for integration of MR into preoperative planning and intraoperative guidance for PCNL have been made.^10^ As MR may potentially improve PCNL outcomes including SFR, puncture time, operative duration and complication rates, we conducted a systematic review and meta‐analysis to assess the clinical applications of MR in PCNL and their influence on patient outcomes.

## 2 METHODS

### 2.1 Search strategy

Using the PICO format, search strategies for Embase, PubMed and Web of Science were designed using Boolean operators to search permutations containing phrases related to AR, endourology and urolithiasis, which can be found in Table S1.

**Population**: adults (≥18 years) undergoing PCNL for urolithiasis.
**Intervention**: MR technologies (AR, VR or MR/fusion navigation) used for preoperative planning and/or intraoperative procedural guidance.
**Comparator**: standard fluoroscopy‐ or US‐guided PCNL without MR‐assistance.
**Outcomes**: SFR (primary); operative duration, puncture time, Clavien–Dindo complications, radiation exposure and length of hospitalization (secondary).

The search strategy was implemented in February 2026 by two authors (Lo CYZ and Toscano F) and in compliance with the Preferred Reporting Items for Systematic Reviews and Meta‐Analyses (PRISMA) guidelines and was registered to the PROSPERO database (registration number CRD420251102638).

### 2.2 Inclusion and exclusion criteria

All studies involving adult patients who underwent PCNL for urolithiasis, in which MR technologies were used for procedural guidance were included in our analysis. All RCTs and nonrandomized comparative studies were included.

Articles primarily focused on PCNL in complex renal anatomy (including but not limited to transplant urolithiasis, renal anatomical variants such as horseshoe kidney and duplex collecting system) were excluded. Systematic reviews, literature reviews, meta‐analyses, letters to the editor, conference abstracts, in vitro studies, animal studies, basic science research and case reports were excluded. Studies published in languages other than English without available translations were also excluded, as translation resources were not available to the review team and reliance on unvalidated translation risked misclassification of interventions and outcomes.

### 2.3 Study selection and data extraction

Titles and abstracts of all studies were independently reviewed by two authors (Lo CYZ and Toscano F) using Rayyan software.^11^ Discrepancies were resolved by discussion between the two authors. This process was repeated for full‐text reviews of all articles selected in the previous stage.

Full‐text articles meeting inclusion criteria underwent data extraction to a prepopulated spreadsheet in Microsoft Excel. The extraction process was independently conducted by two authors (Lo CYZ and Toscano F) and discrepancies were resolved by discussion between the two authors.

### 2.4 Data synthesis and meta‐analysis

Given the emerging nature of MR‐assisted PCNL and the limited number of available RCTs, we included both randomized and nonrandomized studies in the synthesis and meta‐analysis. The primary outcome was the stone‐free rate (SFR). Secondary outcomes were operative duration, renal puncture time, perioperative complications graded by the Clavien–Dindo classification, radiation exposure (fluoroscopy time and/or cumulative dose) and length of hospitalization. For continuous outcomes (puncture time, operative duration and length of hospitalization), we calculated standardized mean differences (SMDs) with 95% confidence intervals (CIs). For dichotomous outcomes suitable for quantitative synthesis, we pooled risk ratios (RRs). Radiation exposure was extracted where reported—fluoroscopy time (seconds or minutes) and/or cumulative dose (mGy) or dose–area product (Gy cm^2^). Owing to heterogeneity in units, measurement methods and reporting protocols, radiation outcomes were summarized narratively rather than pooled quantitatively.

All meta‐analyses were conducted using the *meta* and *metafor* packages in R (version 4.1.1). In cases requiring approximation of mean and standard deviation using other available metrics (e.g., median, range and interquartile range), we utilized an approximation method previously described by Wan et al.^12^ Heterogeneity was quantified using the *I* ^2^ statistic and interpreted according to Cochrane guidelines: 0%–40% might not be important; 30%–60% may represent moderate heterogeneity; 50%–90% may represent substantial heterogeneity; and 75%–100% may indicate considerable heterogeneity. These thresholds were used as a general guide, with interpretation informed by the context and consistency of the results. Subgroup analyses were performed for the following: (1) RCT‐only, (2) PCNL‐only, (3) CT‐imaging‐only studies, and (4) studies with intraoperative MR‐assistance.

Publication bias was assessed visually using funnel plots. Where asymmetry was suspected, Duval and Tweedie's trim‐and‐fill method was applied.

### 2.5 Quality assessment

Randomized controlled trials (RCTs) underwent quality assessment via Cochrane Collaboration's Risk of Bias 2 (RoB2) tool for internal validity. Each study was assigned an overall risk of bias of ‘low’, ‘some concerns’ or ‘high’. Nonrandomized studies were reviewed with the Risk of Bias in Nonrandomized Studies of Interventions (ROBINS‐I) tool. Each study was assigned an overall risk of bias of ‘low’, ‘moderate’, ‘serious’ or ‘critical’. The quality assessment process was independently conducted by two authors (Lo CYZ and Ng JJ), with any discrepancies resolved by discussion between authors. The R tool *robvis* was utilized to create the risk‐of‐bias plots for both RoB2 and ROBINS‐I.^13^

## 3 RESULTS

### 3.1 Study selection

**FIGURE 1:**
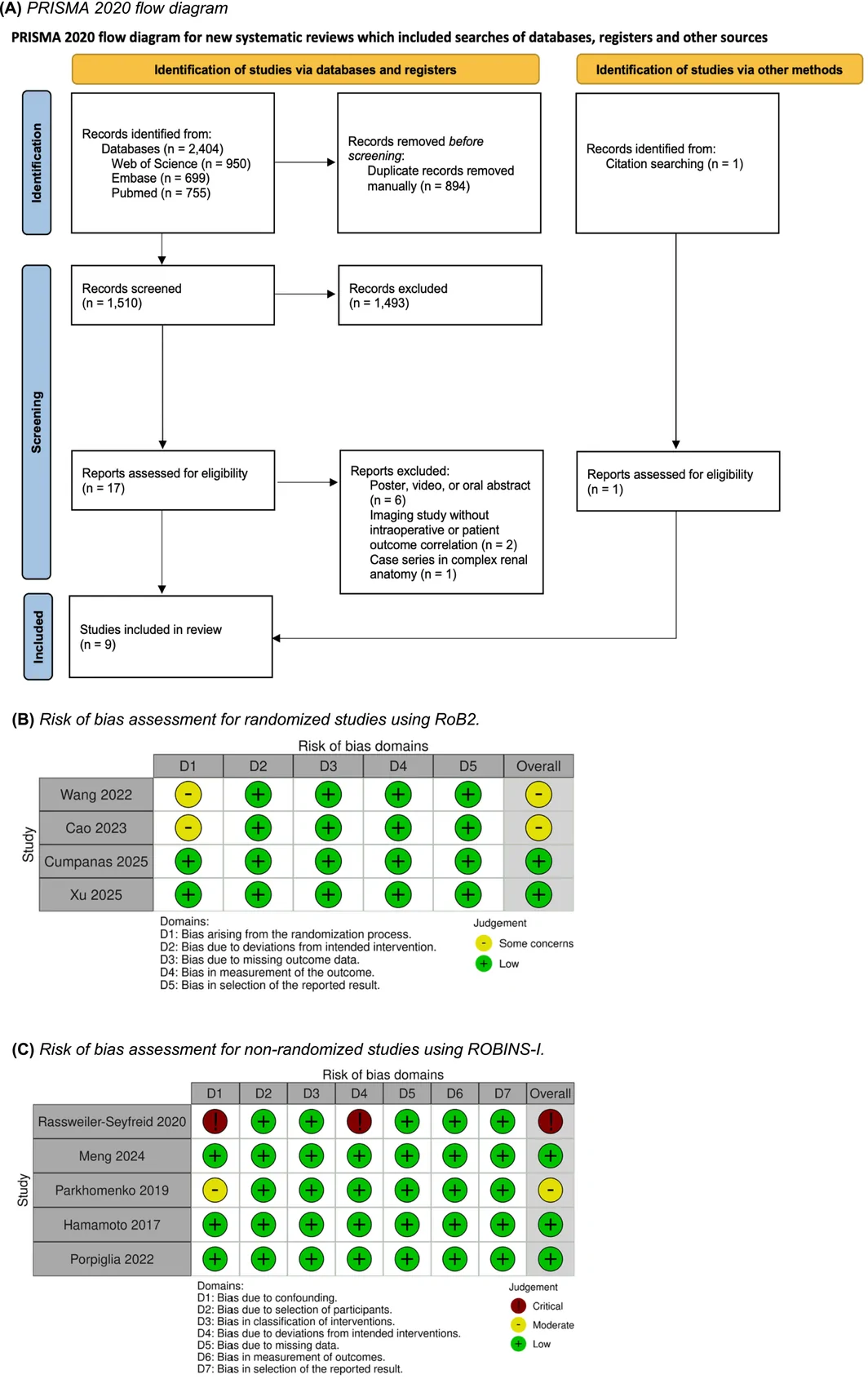
Overview of study selection and risk‐of‐bias assessment. (A) PRISMA 2020 flow diagram. (B) Risk‐of‐bias assessment for randomized studies using RoB2. (C) Risk‐of‐bias assessment for nonrandomized studies using ROBINS‐I.

A total of 2404 records were imported for screening, of which 894 duplicates were manually removed on Rayyan software. One additional article was identified through citation searching. 1510 records underwent title and abstract screening, and 17 full‐text articles were assessed for eligibility. After full‐text review, nine studies were included for qualitative synthesis. Of these, one PCNL paper was ineligible for meta‐analysis in view of the critically assessed risk of bias in confounding and deviations from intended interventions (Figure 1C). The article selection process is summarized in Figure 1A.

### 3.2 Study characteristics

Table 1A provides an overview of MR interventions used across the studies. Most included studies utilized computed tomography (CT)‐reconstructed 3D models, which were then processed and segmented for intraoperative visualization using various platforms. Of the included studies, the following technologies for MR‐assistance were identified: wearable AR devices,^10^, ^14^, ^15^, ^16^, ^17^ tablets,^18^ and CT‐ultrasound (US) fusion navigation.^19^, ^20^ Two studies only used preoperative MR‐assistance without intraoperative guidance,^14^, ^16^ while one study utilized intraoperative AR‐guided needle guidance during PCNL alone without any CT‐reconstructed 3D model.^21^ Baseline characteristics of the study populations are summarized in Table 1B.

**TABLE 1A:** Summary of mixed reality (MR) interventions in included studies.

Author, year	Study design	Procedure	Preoperative MR intervention	Intraoperative MR intervention
Hamamoto, 2017	Non‐RCT	ECIRS	CT reconstruction of 3D model via HI VISION Ascendus software	RVS system to provide cross‐sectional multiplanar reconstructed image
Parkhomenko, 2019	Non‐RCT, matched pair analysis	PCNL	CT reconstruction of 3D model via 3D Slicer software and uploaded to Bosc (iVR software), visualized with Oculus Rift and Touch controllers with Leap Motion display AR	N/A
Rassweiler‐Seyfried, 2020	Non‐RCT, matched pair analysis	PCNL	CT/MRI reconstruction of 3D model via Medical Interaction Tool Kit (MITK)	iPad‐assisted AR
Porpiglia, 2022	Non‐RCT	ECIRS	CT reconstruction of 3D model via Medics software using DICOM viewer and semiautomatic segmentation, hologram setup with Fifthingenium	HoloLens MR
Wang, 2022	RCT	PCNL	CT reconstruction of 3D model and preoperative determination of target calyx for puncture	HoloLens glasses 2 AR
Cao, 2023	RCT	PCNL	CT reconstruction of 3D model via Visual3D software and segmented semiautomatically	MR ophthalmoscope and glasses
Meng, 2024	Non‐RCT	PCNL	CT reconstruction of 3D model and preoperative puncture site selection	Fused navigation system with US MR
Cumpanas, 2025	RCT	PCNL	CT reconstruction of 3D model via 3D Slicer software and manual labelling of intrarenal and perirenal structures by research physician, visualized with Oculus Quest 2 headset AR	N/A
Xu, 2025	RCT	PCNL	N/A	AcuSee AR‐guided US system

Abbreviations: AR, augmented reality; CT, computed tomography; ECIRS, endoscopic combined intrarenal surgery; HI VISION, Hitachi Vision; iVR, immersive virtual reality; MITK, Medical Interaction Toolkit; MR, mixed reality; MRI, magnetic resonance imaging; PCNL, percutaneous nephrolithotomy; RCT, randomized controlled trial; RVS, real‐time virtual sonography; US, ultrasound; VR, virtual reality.

**TABLE 1B:** Baseline characteristics of included studies.

Author, year	Country	Type	*N*	Age, mean (SD)	*p*‐value	Male sex, *n* (%)	*p*‐value	BMI (kg/m2), mean (SD)	*p*‐Value	Stone volume (cm3), mean (SD)	*p*‐Value
Hamamoto, 2017	Japan	I	15	57.5 (3.8)	*p* = 0.89	11 (73.3%)	*p* = 0.45	24.3 (1.3)	*p* = 0.63	3.4 (0.3) [diameter, cm]	*p* = 0.55
		C	15	58.4 (3.9)		8 (53.3%)		24.7 (1.0)		3.1 (0.4) [diameter, cm]	
Parkhomenko, 2019	USA	I	25	55.9 (15.5)	*p* = 0.17	NA	NA	29.5 (5.9)	*p* = 0.22	3.5, 2.9 ** [diameter, cm]	*p* = 0.12
		C	25	61.0 (9.8)		NA		27.1 (8.0)		5.2, 3.9 ** [diameter, cm]	
Rassweiler‐Seyfried, 2020	Germany	I	22	50, 24–83*	NA	NA	NA	26.6, 21–47*	NA	0.15, 0.02–2.86*	NA
		C	22	50.5, 33–83*		NA		25.65, 23.4–44.3*		0.31, 0.04–2.75*	
Porpiglia, 2022	Italy	I	10	56, 54–57 **	*p* = 0.9	6 (60%)	*p* = 0.6	26, 25–27 **	*p* = 0.1	3.5, 3.3–3.5 ** [diameter, cm]	*p* = 0.5
		C	10	56, 54–58 **		5 (50%)		27, 26–28 **		3.7, 3.5–3.8 ** [diameter, cm]	
Wang, 2022	China	I	21	54.0 (10.5)	*p* = 0.14	16 (76.2%)	*p* = 0.61	26.0 (3.0)	*p* = 0.85	4.08 (3.11)	*p* = 0.70
		C	40	58.1 (9.9)		28 (70.0%)		25.8 (3.1)		3.69 (4.18)	
Cao, 2023	China	I	100	47.5 (13.5)	*p* > 0.05	61 (61%)	*p* > 0.05	26.5 (4.5)	*p* > 0.05	8.7 (3.1)	*p* > 0.05
		C	100	45.9 (14.9)		59 (59%)		25.6 (4.5)		7.9 (3.3)	
Meng, 2024	China	I	70	47.7 (10.3)	*p* = 0.66	36 (51.4%)	*p* = 0.60	23.6 (2.2)	*p* = 0.43	5.0 (1.0) [diameter, cm]	*p* = 0.15
		C	66	46.9 (10.7)		31 (47.0%)		23.9 (2.1)		5.2 (0.9) [diameter, cm]	
Cumpanas, 2025	USA	I	86	62, 14**	*p* = 0.23	45 (52%)	*p* = 0.33	30.66 (7.8)	*p* = 0.24	5.39, 1.42–8.97*	*p* = 0.37
		C	89	59, 25**		40 (45%)		29.25 (7.8)		4.91, 1.65–8.09*	
Xu, 2025	China	I	29	55, 27–71***	*p* = 0.92	23 (79%)	*p* = 1.00	25.3 (3.9)	*p* = 0.17	2.52, 1.20–4.50*** [diameter, cm]	*p* = 0.93
		C	29	55, 23–74***		23 (79%)		26.5 (2.9)		2.54, 1.24–4.67*** [diameter, cm]	

Abbreviations: BMI, body mass index; C, control group; I, intervention group; IQR, interquartile range; NA, not available; SD, standard deviation; USA, United States of America.

* median and range.

** median and IQR.

*** mean and range.

Overall, 774 participants were included across the nine studies. Of these, 378 patients underwent MR‐assisted PCNL utilizing technologies such as immersive VR, holographic MR guidance or CT‐US fusion navigation, while 396 patients comprised the control groups that underwent standard fluoroscopy‐ or ultrasound‐guided approaches. Larger sample sizes (>100 total participants) were reported in studies from China^15^, ^19^ and the United States.^14^ Across studies, there were no statistically significant differences between MR‐assisted and standard groups in age, sex, body mass index (BMI) or stone burden.

### 3.3 Key outcomes and complications—Narrative review

Key clinical outcomes are presented in Table 2A. SFR was reported in most studies, which defined SFR as either completely stone‐free or residual fragments ≤4 mm (Table S2). The most common imaging modality for outcome assessment was CT. However, only three studies specified the timing of postoperative imaging.^14^, ^17^, ^20^ Only Cumpanas et al. demonstrated a statistically significant improvement in SFR in the MR‐assisted group compared to controls (63% vs. 47%, *p* < 0.05).^14^

**TABLE 2A:** Key procedural outcomes comparing MR‐assisted and standard procedures.

Author, year	Group	SFR, n (%)	*p*‐Value	Op duration (minutes), mean (SD)	*p*‐Value	Puncture time (minutes), mean (SD)	*p*‐Value	Change in puncture time (I‐C) (minutes)	Number of PCN channels, median (range)	*p*‐Value	Number of puncture attempts per PCN channel, median (range)	*p*‐Value	Success rate of single puncture (%)	*p*‐Value
Hamamoto, 2017	I	13 (87.7%)	*p* = 0.65	102.9 (13.7)	*p* = 0.32	NA			NA		1.6 (0.4) [mean, SD]	***p* = 0.001**	NA	
	C	11 (73.3%)		120.2 (11.5)		NA			NA		3.4 (0.4) [mean, SD]		NA	
Parkhomenko, 2019	I	11/23 (48%)	*p* = 0.16	155, 63.5**	*p* = 0.19	NA			NA		1.13 (0.46) [mean, SD]	*p* = 0.09	NA	
	C	7 (28%)		180, 100**		NA			NA		1.46 (0.78) [mean, SD]		NA	
Rassweiler‐Seyfried, 2020	I	NA		NA		2.65 (1.28)	*p* = 0.55	+0.33	NA		1 (1–4), p = 0.45		63.6% (14/22)	*p* = 0.45
	C	NA		NA		2.32 (1.38)			NA		1 (1–2)		68.2% (15/22)	
Porpiglia, 2022	I	10 (100%)	*p* = 0.30	108, 105–124**	***p* = 0.02**	27, 24–28**	***p* < 0.01**	+12	NA		NA		100% (10/10)	***p* = 0.03**
	C	9 (90%)		91, 90–95**		15, 11–18**			NA		NA		50% (5/10)	
Wang, 2022	I	19 (90.5%)	*p* = 0.19	91.2 (35.0)	*p* = 0.62	8.9 (3.3)	***p* < 0.001**	−5.6	1 (1–3)	*p* = 0.37	1 (1–3)	***p* = 0.03**	NA	
	C	29 (72.5%)		95.8 (33.1)		14.5 (6.1)			1 (1–2)		2 (1–6)		NA	
Cao, 2023	I	97 (97%)	*p > 0.05*	60.7 (10.6)	***p* < 0.001**	6.5 (1.2)	***p* < 0.001**	−2.8	NA		NA		NA	
	C	96 (96%)		68.4 (11.5)		9.3 (4.9)			NA		NA		NA	
Meng, 2024	I	61 (87.1%)	*p* = 0.34	83.8 (16.7)	*p* = 0.60	4.4 (0.7)	***p* = 0.003**	−0.4	1 (1–2)	*p* = 0.65	NA		88.6% (62/70)	***p* = 0.004**
	C	56 (84.8%)		82.4 (14.7)		4.8 (0.8)			1 (1–2)		NA		68.2% (45/66)	
Cumpanas, 2025	I	54 (63%)	***p* = 0.04**	142 (51)	*p* = 0.24	NA			NA		1 (1–5)**	*p* = 0.86	NA	
	C	43 (47%)		152 (60)		NA			NA		1 (1–4)**		NA	
Xu, 2025	I	23 (79%)	*p* = 0.55	83, 29–122***	*p* = 0.27	3.12, 0.20–6.88 ***	***p* < 0.001**	−4.46	NA		1, 1–3 * [total attempts]	*p* = 0.47	86.2% (25/29)	NA
	C	20 (69%)		92, 38–200 ***		7.58, 5.41–10.68 ***			NA		1, 1–2 * [total attempts]		93.1% (27/29)	

*Note*: SFR was available for 23 out of 25 participants in the intervention arm (Parkhomenko).

Abbreviations: I, intervention group; C, control group; IQR, interquartile range; NA, not available; Op, operative; PCN, percutaneous nephrostomy; SD, standard deviation; SFR, stone‐free rate.

* median and range.

** median and IQR.

*** mean and range.

For operative duration, most studies did not find significant differences; however, Cao et al. reported a statistically significant reduction in operative time for the MR group (60.7 vs. 68.4 min, *p* < 0.001).^15^

Across the six studies reporting puncture time (Table 2A), the mean difference between MR‐assisted and control groups ranged from a reduction of 5.6 min (Wang et al.) to an increase of 12 min (Porpiglia et al.).^10^, ^17^ Puncture time was shorter with MR‐assistance in four studies (Wang −5.6 min, Xu −4.46 min, Cao −2.8 min and Meng −0.4 min)^10^, ^15^, ^19^, ^21^ and longer in two (Porpiglia +12 min; Rassweiler‐Seyfried +0.33 min).^17^, ^18^ The direction and magnitude of effect varied by platform: CT‐US fusion systems^15^, ^19^ shortened puncture time, whereas headset/holographic overlays were mixed (shorter with Wang's HoloLens, longer with Porpiglia's despite 100% first‐pass success), and iPad‐based AR (Rassweiler‐Seyfried) slightly increased it.^10^, ^17^, ^18^ No difference in the number of PCN tracts was observed across studies.^10^, ^19^

Radiation exposure was inconsistently reported across studies, expressed as fluoroscopy time and/or cumulative dose. Because of nonuniform units and imaging protocols, no meta‐analysis was performed. Directionally, fusion‐based MR and CT‐US navigation systems tended to reduce fluoroscopy duration, whereas tablet‐based MR platforms showed no difference or, in isolated cases, slightly increased exposure. Porpiglia et al. found MR guidance was associated with statistically significant reductions in median radiation exposure time of 138 s (95% CI: −205.3 to −70.6, *p* < 0.01).^17^

While Wang et al.^10^ found a statistically significant reduction in the number of puncture attempts per tract when using MR technologies (*p* = 0.03), other studies did not demonstrate this benefit.^14^, ^16^, ^21^ iPad‐assisted AR did not improve single puncture success rates.^18^ Instead, CT‐US fusion navigation and MR HoloLens systems reported statistically significant improvements (*p* = 0.004 and *p* = 0.03, respectively).^17^, ^19^

#### 3.3.1 Complications

Use of MR was associated with either reduced or comparable rates of complications (Table 2B). Several studies^10^, ^15^, ^17^, ^20^, ^21^ reported zero Clavien–Dindo Grade 3 or 4 complications in the MR‐assisted group. Commonly reported Grade 1 and 2 complications included hematuria, fever, urinary retention and urinary tract injury. Reported Grade 3 and 4 events included haemorrhages requiring angioembolization, septic shock, pleural effusions requiring drainage, postoperative DJ stent repositioning and ureteral stenosis. Of the studies that reported individual complication rates,^10^, ^14^, ^16^, ^17^, ^20^, ^21^ 0/186 cases of pleural effusion were reported in the MR‐assisted group, while 5/208 (2.4%) cases in the control arm required chest drain insertion for pleural effusion (Clavien–Dindo 3a). For the same studies, 3/186 (1.6%) cases of haemorrhage requiring angioembolization were reported in the MR‐assisted group compared to 8/208 (3.8%) in the control group. The low event rates of Grade 3 events such as haemorrhage requiring angioembolization and pleural effusions requiring drainage precluded further quantitative analysis. Across included studies, there was no statistically significant difference in length of hospitalization between MR‐assisted and control groups.^15^, ^17^, ^19^, ^20^

**TABLE 2B:** Complications and length of hospitalization.

Author, year	Group	*N*	Clavien–Dindo complications				Type of complications	Length of hospitalization (days), mean (SD)	*p*‐Value
			1	2	3	4			
Hamamoto, 2017	I	15	2	1	0	0	1: Transient fever >38.5°C, urinary tract injury	4.9 (3.3)	*p* = 0.95
	C	15	1	1	1	1	1: Transient fever >38.5°C, urinary tract injury 2: Blood transfusion 3: Renal haemorrhage requiring angioembolization 4: Sepsis	5.0 (0.7)	
Parkhomenko, 2019	I	25	3 (12%)	0	1 (4%)	0	3: Pneumothorax	NA	
	C	25	1 (4%)	0	0	0	NA	NA	
Rassweiler‐Seyfried, 2020	I	22	0	0	0	0	NA	NA	
	C	22	0	0	0	0	NA	NA	
Porpiglia, 2022	I	10	3 (30%)		0 (0%)		1 + 2: 2× Hematuria, 1× fever	4, 4–5 (median, IQR)	*p* = 0.06
	C	10	3 (30%)		1 (10%)		1 + 2: 2× Hematuria, 1× urinary retention 3 + 4: Ureteral stenosis	4, 3–4 (median, IQR)	
Wang, 2022	I	21	5 (23.8%)	2 (9.5%)	0	0	NA	NA	
	C	40	16 (40%)	3 (7.5%)	3 (7.5%)	1 (2.5%)	3: 1× Postoperative DJ stent position adjustment, 1× catheter compression of percutaneous renal channel bleeding, 1× pleural effusion drainage 4: Septic shock	NA	
Cao, 2023	I	100	**12 (12%)**	**0 (0%)**	**0 (0%)**		NA	4.0 (2.0)	*p* > 0.05
	C	100	**21 (21%)**	**1 (1%)**	**1 (1%)**		NA	4.5 (2.0)	
Meng, 2024	I	70	6		NA	NA	NA	8.5 (1.5)	*p* = 0.13
	C	66	5		NA	NA	NA	8.1 (1.5)	
Cumpanas, 2025	I	86	8 (9%)	**3 (3%), *p* = 0.03**		NA	2 + 3a: 3× Haemorrhages requiring angioembolization	NA	
	C	89	6 (7%)	**11 (12%)**		NA	2 + 3a: 5× Haemorrhages requiring angioembolization, 4× pleural effusion requiring chest drain placement, 2× urosepsis events	NA	
Xu, 2025	I	29	**2 (7%)**	**1 (3%)**	**0**	0	NA	NA	
	C	29	**6 (21%)**	**3 (10%)**	**3 (10%)**	0	3a: 2× haemorrhages requiring angioembolization, 1× displaced DJ tube after surgery	NA	

Abbreviations: C, control group; DJ, double‐J; I, intervention group; IQR, interquartile range; NA, not available; SD, standard deviation.

#### 3.3.2 Publication bias

The funnel plot for SFR (Figure S1A) demonstrated asymmetry, with most studies distributed to the right of the pooled estimate and few smaller studies favouring the control arm. Duval and Tweedie trim‐and‐fill (Figure S1B) imputed studies on the left side of the distribution and shifted the adjusted estimate toward the null, suggesting the borderline pooled SFR result may be influenced by small‐study effects. Interpretation is limited, however, as with fewer than 10 studies trim‐and‐fill is underpowered and the observed asymmetry may partly reflect between‐study heterogeneity rather than publication bias alone. Residual small‐study effects therefore cannot be excluded, and the SFR finding should be regarded as hypothesis‐generating.

### 3.4 Meta‐analysis results

Across the eight studies included in the quantitative synthesis, a total of 730 participants were analysed, comprising 356 patients in the MR‐assisted intervention arms and 374 in the control arms. The MR‐assisted interventions included immersive VR preoperative planning, intraoperative MR holographic guidance, CT‐US fusion navigation systems and intraoperative AR‐guided US systems for needle placement. Pooled analyses were conducted for the primary outcomes of SFR, operative duration and puncture time, with each outcome evaluated using random‐effects models to account for expected heterogeneity across populations and MR platforms. Subgroup analyses for RCT‐only, PCNL‐only studies, studies utilizing CT imaging for SFR and studies incorporating intraoperative MR‐assistance can be found in the Figures S2–S5.

**FIGURE 2:**
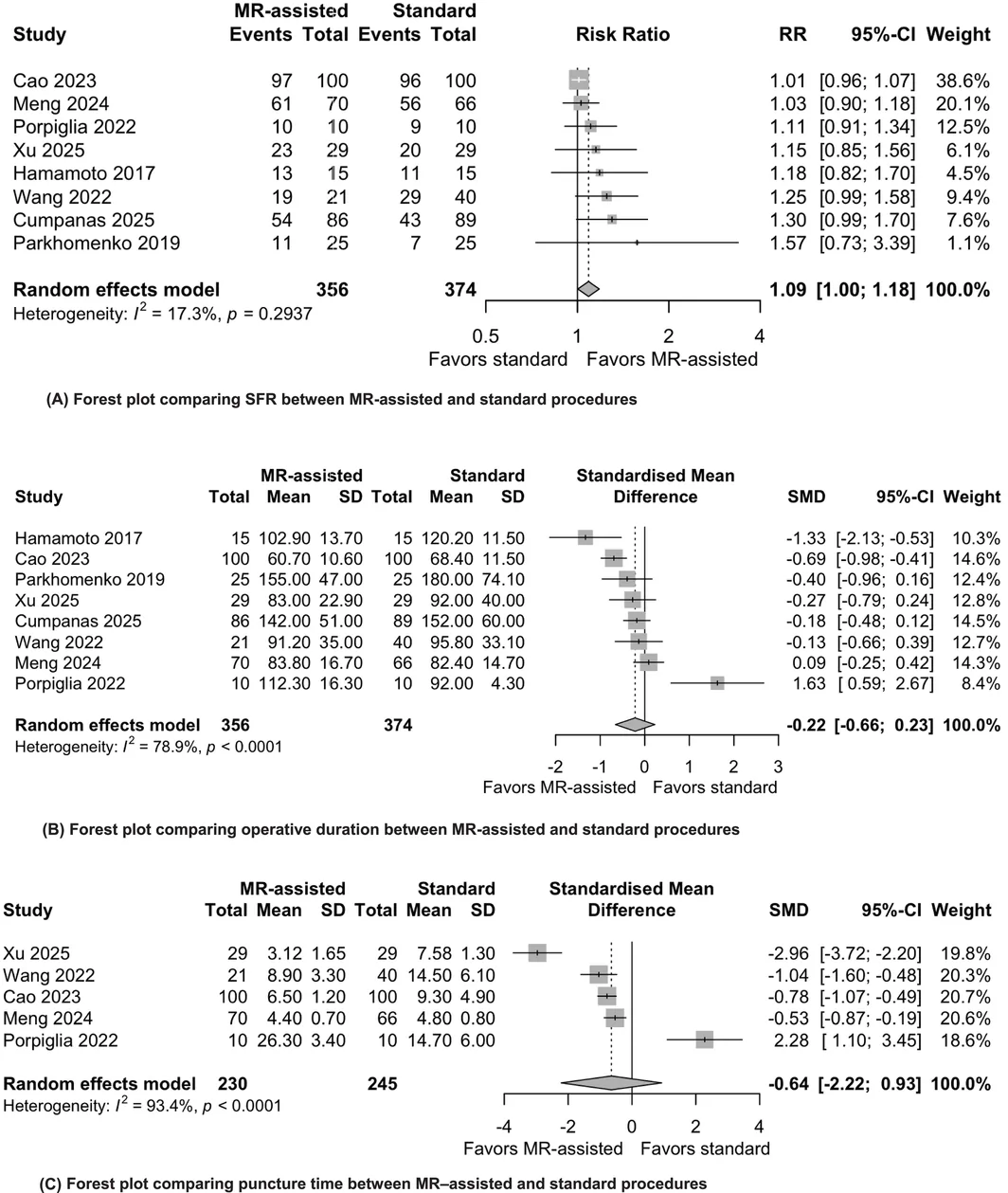
(A) Forest plot comparing SFR between MR‐assisted and standard procedures. (B) Forest plot comparing operative duration between MR‐assisted and standard procedures. (C) Forest plot comparing puncture time between MR–assisted and standard procedures.

Meta‐analysis of the eight studies evaluating SFR yielded a pooled RR of 1.09 [95%CI 1.00–1.18, *p* = 0.044], suggesting a modest difference between MR‐assisted and standard approaches (Figure 2A), with low observed heterogeneity (*I* ^2^ = 17.3%, *p* = 0.29). Eight studies evaluating operative duration had a pooled SMD of −0.22 (95%CI‐0.66 to 0.23, *p* = 0.34), which was not statistically significant with high heterogeneity (*I* ^2^ = 78.9%, *p* < 0.0001) (Figure 2B). Five studies reported puncture time, of which pooled SMD‐0.64 [95%CI‐2.22 to 0.93, *p* = 0.42], which was also not statistically significant and had considerable heterogeneity (*I* ^2^ = 93.4%, *p* < 0.0001) (Figure 2C).

#### 3.4.1 PCNL‐only studies

Subgroup analysis of SFR using six PCNL‐only studies found a pooled RR of 1.09 (95% CI 0.99–1.21, *p* = 0.088), which was not statistically significant (Figure S2A). Heterogeneity was low (*I* ^2^ = 33.7%, *p* = 0.18). Subgroup analysis of operative duration using six PCNL‐only studies had a pooled SMD of −0.27 (95% CI‐0.53 to −0.02, *p* = 0.038), which was a statistically significant difference between MR‐assisted and standard PCNL (Figure S2B), with moderate heterogeneity (*I* ^2^ = 63.1%, *p* = 0.019). Subgroup analysis of puncture time using PCNL‐only studies noted a pooled SMD −1.29 (95% CI‐2.33 to −0.25, *p* = 0.015), indicating a statistically significant reduction in puncture time in the MR‐assisted group; however heterogeneity was considerable (*I* ^2^ = 91.1%, *p* < 0.0001) (Figure S2C).

#### 3.4.2 RCT‐only studies

Subgroup analysis of SFR using four RCT‐only studies demonstrated a pooled RR of 1.13 (95% CI 0.98–1.30, *p* = 0.10), which was not statistically significant (Figure S3A). Heterogeneity was moderate (*I* ^2^ = 53.1%, *p* = 0.094). Subgroup analysis of operative duration using four RCT‐only studies had a pooled SMD −0.35 (95% CI −0.65 to −0.06, *p* = 0.019), which suggested a statistically significant difference between MR‐assisted and standard approaches with moderate heterogeneity (*I* ^2^ = 59.4%, *p* = 0.061) (Figure S3B). Subgroup analysis of puncture time using three RCT‐only studies noted a pooled SMD −1.56 (95% CI −2.88 to −0.24, *p* = 0.021), indicating a statistically significant reduction in puncture time (Figure S3C). Heterogeneity was considerably high (*I* ^2^ = 92.8%, *p* < 0.0001).

#### 3.4.3 Studies utilizing CT imaging for SFR definition

Subgroup analysis of SFR included six studies that utilized CT imaging for detecting residual stones demonstrated a pooled RR of 1.13 (95% CI 1.02–1.25, *p* = 0.017), which was statistically significant (Figure S4A). Observed heterogeneity was low, albeit with a wide confidence interval (*I* ^2^ = 0.0%, *p* = 0.50, 95% CI 0.0%–74.6%).

#### 3.4.4 Intraoperative MR‐assisted studies

Subgroup analysis of SFR using six intraoperative MR‐assisted‐only studies demonstrated a pooled RR of 1.05 (95% CI 0.98–1.12, *p* = 0.15), which was not statistically significant (Figure S5A). Similarly to the CT‐imaging subgroup, heterogeneity was low with a wide confidence interval (*I* ^2^ = 0.0%, *p* = 0.47, 95% CI 0.0%–74.6%). Subgroup analysis of operative duration using six intraoperative MR‐assisted‐only studies had a pooled SMD −0.17 (95% CI −0.85 to 0.51, *p* = 0.63), which was not statistically significant (Figure S5B). Heterogeneity was significant (*I* ^2^ = 84.5%, *p* < 0.0001). Subgroup analysis of puncture time was not performed as the included studies in the main analysis were identical to this subgroup.

### 3.5 Risk‐of‐bias assessment

Of the included RCTs, Wang et al.^10^ and Cao et al.^15^ lacked information regarding concealment of allocation sequence. Other domains were assessed to have low risk of bias. With respect to non‐RCTs, most studies had low risk of bias. However, Rassweiler‐Seyfried et al.^18^ were assessed to have critical risk of bias in domains of confounding and possible deviations from intended interventions. In their study, reported technical difficulties precluded analysis of 10/22 of their participants. Furthermore, key information regarding patient baseline characteristics was missing, such as patient sex, stone laterality and dimension(s). In Parkhomenko's study,^16^ some concerns for risk of bias were assigned in view of incomplete reporting of stone position and measures to monitor and adjust for varying surgeon experience with endourological procedures. A summary of risk‐of‐bias judgements across all included studies is provided in Figure 1B,C.

## 4 DISCUSSION

This systematic review and meta‐analysis found that MR‐assisted PCNL was associated with a small, borderline increase in SFR. This result was not robust across subgroup analyses and may be influenced by small‐study effects. Operative duration and puncture time remained comparable to conventional approaches. Improvements in bleeding and radiation metrics were observed in several individual studies, although heterogeneity in reporting limited pooled interpretation. The following sections examine the diversity of MR platforms, their functional roles within the surgical workflow and how these outcome‐specific findings align with current evidence and operator experience.

### 4.1 Spectrum of MR modalities

Table 1A summarizes the spectrum of MR technologies evaluated in clinical studies of PCNL. Across the eight studies, preoperative interventions were based on CT‐derived 3D reconstructions, created through segmentation software such as 3D Slicer, Visual3D, MITK or Medics. These reconstructions were then applied in different ways depending on the platform: immersive VR for preoperative planning and rehearsal, AR overlays for intraoperative puncture alignment using tablets or headsets, MR systems combining holographic visualization with US fusion and real‐time virtual sonography (RVS) synchronizing cross‐sectional CT with intraoperative US. Intraoperative implementations ranged from marker‐based AR overlays^18^ and holographic guidance with HoloLens^10^, ^17^ to fusion‐based navigation systems.^15^, ^19^, ^20^ Collectively, these studies illustrate both the diversity of available MR modalities and the evolving attempts to integrate them at different points of the surgical workflow, from preoperative rehearsal to live intraoperative access guidance.

### 4.2 Functional roles of MR platforms

The comparative evidence highlights that these modalities fulfill distinct roles depending on when and how they are deployed. Immersive VR has been used primarily as a preoperative adjunct, enabling surgeons to rehearse access tracts, select the optimal calyx and develop a more intuitive understanding of complex or anomalous anatomy. In a randomized trial, Cumpanas et al.^14^ showed that immersive rehearsal altered tract selection in nearly a third of cases and improved SFR with fewer complications compared to CT planning alone, while Parkhomenko et al.^16^ reported reduced fluoroscopy time and blood loss in a pilot series.

In contrast, intraoperative overlays such as iPad‐based AR or HoloLens holograms provide real‐time visual alignment during renal puncture. Porpiglia et al.^17^ demonstrated that HoloLens‐based holograms achieved 100% first‐pass success and reduced radiation, albeit with longer puncture times; although Rassweiler‐Seyfried et al.^18^ found that iPad‐assisted AR increased puncture time and radiation exposure compared to standard access.

Fusion‐based MR systems have produced the most consistent intraoperative benefits. Cao et al. demonstrated in a large, randomized trial that MR‐assisted puncture shortened access time, reduced operative duration and minimized blood loss relative to US guidance alone.^15^ Hamamoto et al. observed fewer puncture attempts and smaller haemoglobin drops with CT‐US fusion guidance specifically in PCNL with ECIRS.^20^ Meng et al. further showed that deep learning‐enhanced MR navigation improved single‐pass success and reduced blood loss in a retrospective series (*n* = 136), particularly benefiting novice operators.^19^

These findings suggest a pragmatic hierarchy: immersive VR as a preoperative rehearsal tool, AR holographic overlays as emerging intraoperative visual aids, and CT‐US fusion systems as the most mature real‐time navigation platforms.

### 4.3 Quantitative outcomes

Pooled analyses demonstrated a modest increase in SFR for MR‐assisted over standard procedures, which was statistically significant (*p* = 0.044). In subgroup analysis of studies utilizing CT imaging for SFR determination, the pooled results still favoured MR‐assistance (*p* = 0.017). However, in the subgroup analysis using RCT‐only, PCNL‐only, as well as studies with both preoperative and intraoperative MR‐assistance, the change in SFR was not statistically significant. These results should be interpreted cautiously and highlight the need for high‐quality RCTs with robust study design and data collection. The included MR technologies in our study predominantly focused on identifying anatomical structures and accurate identification of the renal stone for optimal puncture site and number. However, their intraoperative utility for detecting residual fragments is not well described. In future, AI technologies for fragmentation detection and video point tracking may be a useful intraoperative aid to further improve SFR.^22^

MR‐assisted procedures did not demonstrate a statistically significant improvement in operative duration with substantial heterogeneity. Subgroup analysis of operative duration in studies utilizing preoperative and intraoperative MR‐assistance did not reach statistical significance. In the subgroup analysis of PCNL‐only studies, operative duration had a small, statistically significant reduction with moderate heterogeneity (SMD −0.27, *p* = 0.038), which is expected as the retrograde approach for stone clearance in ECIRS requires setup time and may increase surgical duration. Longer operative times have been linked to higher rates of severe complications (e.g., Clavien–Dindo Grade III–IV) in a large‐scale series of PCNL patients.^23^ Currently, MR technologies are still in their early stages of development and clinical implementation, which may introduce significant variations to intraoperative temporal parameters as there is setup time required for the MR glasses, registering the fusion navigation system, and intraoperative troubleshooting.

Puncture time had a nonsignificant improvement and was subject to a high degree of heterogeneity. However, subgroup analysis of PCNL‐only studies found MR integration reduced puncture time, suggesting that the use of augmented visualization and navigation facilitates quicker identification and access to targeted renal structures. This advantage likely stems from enhanced spatial orientation and confidence provided by 3D visualization, leading to fewer puncture attempts and improved precision.^10^, ^19^ Other studies demonstrated that CT‐US fusion guidance improved puncture accuracy, particularly in anatomically challenging cases, reinforcing the potential of MR to enhance procedural efficiency.^20^

### 4.4 Stone complexity

Heterogeneity in stone complexity may partly explain the variability in observed MR benefit. Cao et al. had the largest mean stone volume (8.7 cm^3^) and reported the most consistent procedural gains including reduced operative duration and puncture time.^15^ In high‐burden or anatomically challenging cases, the spatial orientation and 3D depth perception afforded by MR could plausibly compensate for the limitations of two‐dimensional fluoroscopic guidance, whereas for straightforward lower pole calculi, conventional access is already reliable. Notably, Cumpanas et al. reported that immersive VR planning altered tract selection in nearly a third of cases, suggesting that preoperative MR has the greatest impact precisely when anatomy is sufficiently complex to warrant reconsideration of the access strategy.^14^ In a case series of 15 patients with complex upper urinary stones, MR‐assisted PCNL achieved 80% SFR, operative duration of 61.5 min (SD ± 12.2), and no Clavien–Dindo grade ≥II complications.^24^ These observations suggest that pooling outcomes across the full complexity spectrum may distort a clinically meaningful signal. Prospective stratification by stone complexity should be a prespecified component of future trial designs.

### 4.5 Bleeding metrics and reporting variability

In terms of bleeding outcomes, the reporting across studies varied considerably. Cao et al. reported estimated blood loss (EBL) between groups alone, while other studies used perioperative haemoglobin changes as a more objective surrogate of intraoperative blood loss.^15^, ^17^, ^19^, ^20^ As EBL may be influenced by the volume of irrigation solution used intraoperatively, we recommend the use of haemoglobin drop perioperatively as an objective measure of blood loss.

### 4.6 Influence of surgical experience

During our analysis, we noted that some authors studied the impact of operator PCNL experience on utilization and perceived benefit of MR guidance. Across studies, the perceived benefit of MR indeed varied by operator experience. Junior urologists frequently reported greater value during puncture and access, where intraoperative visual augmentation supported spatial orientation and real‐time anatomy.^17^, ^20^ Senior surgeons, while less dependent on MR during execution, still recognized advantages for surgical planning and workflow. In some cases, experienced clinicians highlighted MR's role in rehearsing anatomically complex cases,^10^, ^19^ while other studies noted that immersive VR planning was used mainly by senior surgeons to map difficult accesses preoperatively.^14^ Overall, MR's utility appears experience‐dependent: Trainees use it as a scaffold for confidence and visual guidance, whereas senior surgeons employ it as an adjunct for planning, efficiency and teaching rather than real‐time navigation.

### 4.7 Implications for training and simulation

The integration of MR technologies into endourology training aligns with a broader pedagogical shift toward immersive, simulation‐based education that prioritizes patient safety and skill acquisition outside the operating room. Both the EAU and AUA have emphasized simulation in their training curricula, yet the incorporation of advanced MR modalities remains inconsistent. Simulation‐based education in urology has largely focused on physical models, animal labs and standard virtual reality simulators, with limited penetration of real‐time MR‐assisted guidance systems.^25^ Ritchie et al. offered a timely update, underscoring that while laparoscopic and robotic simulators have matured, simulation in percutaneous access and fluoroscopy‐guided procedures remains underdeveloped.^26^ We suggest further exploration of MR integration for a dual purpose: to improve operative outcomes and provide a scaffold for the residents' PCNL learning curve.

## 5 LIMITATIONS

This review is subject to several important limitations. First, there was substantial heterogeneity in the design and implementation of MR technologies. The included studies employed a broad spectrum of hardware platforms (e.g., iPad and HoloLens), software tools and surgical applications, which limits pooled interpretation. As previously mentioned, two studies utilized MR‐assistance for preoperative planning and rehearsal alone,^14^, ^16^ compared to the remaining studies that also incorporated intraoperative MR‐assistance for active navigation, which may limit the generalizability of our findings. Despite this, the included studies' technologies were all designed to enhance 3D spatial understanding and guidance. Furthermore, these technologies are all functionally related within the same class of intervention. Hence, our results were interpreted in the context of the overall effect of MR‐assistance.

Technical variables, such as tract size, imaging guidance (US vs. fluoroscopy only) and specific procedural approaches such as ECIRS were not consistently reported and remain unadjusted in the current pooled analysis. In future, adequately powered standardized randomized trials for MR‐assisted PCNL are recommended to interrogate key outcome measures such as SFR, fluoroscopy time, puncture time, severe complications such as bleeding requiring angioembolization and pleural effusion.

Outcome definitions such as SFR lacked standardized reporting across studies, which may have introduced a risk of misclassification and reduced comparability. Although most studies adopted a ≤4‐mm residual fragment threshold,^10^, ^14^, ^17^, ^21^ others such as Rassweiler‐Seyfried et al.^18^ did not specify a numerical cutoff, and Cao et al.^15^ lacked detail on fragment size cutoff despite using CT imaging for residual stone detection (Table S2). Additionally, the timing and imaging modality used to assess SFR varied or were not consistently reported. Only a few studies, such as Hamamoto et al. explicitly stated the postoperative time point (i.e., 4 weeks).^20^ While CT is considered the gold standard due to its high sensitivity, several studies relied on US and abdominal X‐ray (AXR), which are less sensitive but more accessible. As such, the observed effects on SFR should be interpreted cautiously. Standardized definitions, imaging protocols and timepoints are essential to ensure consistency and reproducibility in future trials. We recommend defining SFR as the use of noncontrasted CT imaging with a ≤4mm residual fragment threshold.

Reporting of key secondary outcomes, such as EBL and radiation exposure, was inconsistent and precluded pooled analysis. Fluoroscopy time was the most commonly reported surrogate, but units varied and measurement start and endpoints were rarely defined, and cumulative dose or dose–area product was reported in only a minority of studies. Standardized metrics should be uniformly reported in future MR‐assisted PCNL trials to facilitate comparability.

Also, none of the included studies performed prespecified subgroup analyses stratified by stone complexity. Factors including but not limited to stone burden, staghorn morphology and the presence of anatomical anomalies such as horseshoe kidney and calyceal diverticulum are known to influence puncture difficulty and subsequent outcomes and therefore may also influence the benefits conferred by MR‐assistance. Future RCTs should therefore be adequately powered to include subgroup analyses by stone complexity to determine which patient subgroups derive the greatest benefit from MR‐assisted PCNL.

Finally, none of the included studies reported long‐term outcomes such as stone recurrence or reintervention rate; the present synthesis is therefore confined to perioperative and short‐term procedural endpoints, and the durability of any MR‐associated benefit remains unknown.

## 6 CONCLUSION

This systematic review and meta‐analysis suggests that MR technologies in PCNL may be associated with a statistically significant increase in SFR, whereas overall operative and puncture times were not significantly different from standard approaches. Although improvements in operative duration and puncture time were observed in some individual studies, these findings were not statistically significant in pooled analysis. To establish its role in routine clinical practice, future studies should adopt standardized protocols, consistent outcome definitions and adequately powered randomized trials. Such trials should be sufficiently powered to facilitate prespecified subgroup analyses stratified by stone complexity to identify which patient populations benefit most from MR guidance. Beyond procedural metrics, MR holds broader potential in surgical education. As MR technologies mature, their integration into clinical workflows may not only enhance technical performance but also contribute to safer, more patient‐centred surgical care. As evidence accumulates, MR is likely to serve as an adjunct—rather than a replacement—within the evolving toolkit of image‐guided endourology.

## AUTHOR CONTRIBUTIONS

**Christopher Yong‐Zyn Lo:** Protocol/project development, data collection or management, data analysis, manuscript writing/editing. **Frida Toscano:** Protocol/project development, data collection or management, data analysis, manuscript writing/editing. **John Joson Ng:** Data collection or management, data analysis, manuscript writing/editing. **Du Jingzeng:** Manuscript writing/editing. **Archan Khandekhar:** Manuscript writing/editing. **Hemendra Shah:** Manuscript writing/editing. **Robert Marcovich:** Manuscript writing/editing. **Julio Ojalvo:** Manuscript writing/editing. **Sarvesh Saini:** Manuscript writing/editing. **Ubbo Visser:** Methodological oversight, critical feedback, manuscript writing/editing. **Jonathan Katz:** Protocol/project development, manuscript writing/editing.

## CONFLICT OF INTEREST STATEMENT

The author declares no conflicts of interest.

## Supporting information


**Figure S1A.** Funnel plot for publication bias assessment of stone‐free rate (SFR).
**Figure S1B.** Trim‐and‐fill analysis for stone‐free rate (SFR).
**Figure S2A‐C.** Forest plots for SFR for PCNL‐only studies.
**Figure S3A‐C.** Forest plots for RCT‐only studies.
**Figure S4.** Forest plots for studies using CT‐imaging for SFR determination.
**Figure S5.** Forest plots for studies utilizing intraoperative MR‐assistance.
**Table S1.** Search strategy.
**Table S2.** SFR definitions.

## Data Availability

All analysed data are publicly available from previously published articles.
