# Correction to “Mixed reality applications in percutaneous stone surgery: a systematic review and meta‐analysis of stone‐free rate and perioperative complications”

**DOI:** 10.1002/bco2.70300

**Published:** 2026-10-22

Lo CY‐Z , Toscano F , Ng JJ , Jingzeng D , Khandekhar A , Shah H , et al. Mixed reality applications in percutaneous stone surgery: A systematic review and meta‐analysis of stone‐free rate and perioperative complications. *BJUI*
 Compass. 2026; 7(9):e70278. 10.1002/bco2.70278 PMC1359339642774022

In the originally published article, the author Ubbo Visser was inadvertently omitted from the author list. Ubbo Visser should have been listed as the tenth (second to last) author, between Sarvesh Saini and Jonathan Katz, with the affiliation Department of Computer Science, University of Miami, Coral Gables, Florida, USA.

The correct author list is as follows:

Christopher Yong‐Zyn Lo, Frida Toscano, John Joson Ng, Du Jingzeng, Archan Khandekar, Hemendra Shah, Robert Marcovich, Julio Ojalvo, Sarvesh Saini, Ubbo Visser, Jonathan Katz.

The online version of this article has been corrected accordingly.

We apologize for this error.

