# Supplementary material for: Mixed reality applications in percutaneous stone surgery: A systematic review and meta‐analysis of stone‐free rate and perioperative complications
Source: BJUI Compass. 2026 Sep 21;7(9):e70278. doi: 10.1002/bco2.70278 (PMC13593396; doi:10.1002/bco2.70278)
Supplement: Supplementary file 1 [file BCO2-7-e70278-s001.docx]

**Supplementary Figure 1A. Funnel plot for publication bias assessment of stone-free rate (SFR).**

**Supplementary Figure 1B. Trim-and-fill analysis for stone-free rate (SFR).**

**Supplementary Figure 2A-C. Forest plots for SFR for PCNL-only studies**

*(A) SFR*

*(B) Operation duration*

*(C) Puncture time*

**Supplementary Figure 3A-C. Forest plots for RCT-only studies**

*(A) SFR*

*(B) Operation duration*

*(C) Puncture time*

**Supplementary Figure 4. Forest plots for studies using CT-imaging for SFR determination**

*(A) SFR*

*(B) Operation duration*

*(C) Puncture time*

**Supplementary Figure 5. Forest plots for studies utilizing intraoperative MR-assistance**

*(A) SFR*

*(B) Operation duration*

| **Database** | **Search Terms** |
| --- | --- |
| PubMed | (("computer vision"[All Fields] OR "virtual reality"[All Fields] OR "augmented reality"[All Fields] OR "mixed reality"[All Fields] OR "hololens"[All Fields])  AND  ("urology"[All Fields] OR "endourology"[All Fields] OR "kidney stone"[All Fields] OR "urolithiasis"[All Fields] OR "nephrolithiasis"[All Fields]))  AND  (english[Filter]) |
| Embase | ( 'computer vision'/exp OR 'computer vision':ti,ab,kw  OR 'virtual reality'/exp OR 'virtual reality':ti,ab,kw  OR 'augmented reality'/exp OR 'augmented reality':ti,ab,kw  OR 'mixed reality':ti,ab,kw  OR 'hololens':ti,ab,kw)  AND  ( 'urology'/exp OR 'endourology'/exp  OR 'kidney stone'/exp OR 'urolithiasis'/exp OR 'nephrolithiasis'/exp  OR urology:ti,ab,kw  OR endourology:ti,ab,kw  OR 'kidney stone*':ti,ab,kw  OR urolithiasis:ti,ab,kw  OR nephrolithiasis:ti,ab,kw)  AND  [english]/lim |
| Web of Science | **(**ALL=(computer vision) OR ALL=(virtual reality) OR ALL=(augmented reality) OR ALL=(mixed reality) OR ALL=(hololens)) AND (ALL=(urology) OR ALL=(endourology) OR ALL=(kidney stone) OR ALL=(urolithiasis) OR ALL=(nephrolithiasis)) |

**Supplementary Table 1. Search strategy**

| **Author, Year** | **Procedure** | **SFR definition** |  |
| --- | --- | --- | --- |
| Hamamoto, 2017 | ECIRS | Absence of residual symptomatic fragments > 4mm diameter at 4-week US kidneys and AXR |  |
|  |  |  |  |
| Parkhomenko, 2019 | PCNL | Categorized as either complete stone free or residual fragments < 4mm on post-operative CT |  |
|  |  |  |  |
| Rassweiler-Seyfried, 2020 | PCNL | NA |  |
|  |  |  |  |
| Porpiglia, 2022 | ECIRS | Success rate defined as stone-free rate and absence of clinically insignificant residual fragment =< 4mm at 3-month CT scan |  |
|  |  |  |  |
| Wang, 2022 | PCNL | Absence of stone fragments > 4mm diameter on post-operative CT |  |
|  |  |  |  |
| Cao, 2023 | PCNL | NA |  |
|  |  |  |  |
| Meng, 2024 | PCNL | Absence of stone fragments > 4mm diameter on post-operative AXR and CT imaging |  |
|  |  |  |  |
| Cumpanas, 2025 | PCNL | Grade A: no stone fragment, Grade B: =< 2mm fragments, Grade C: 2.1-4mm fragments within 3-month CT scan |  |
|  |  |  |  |
| Xu, 2025 | PCNL | Stone clearance was defined as clinically insignificant stone fragments of ≤ 4 mm obtained by post-operative renal, ureteral, and bladder CT scans |  |
|  |  |  |  |

**AXR, abdominal X-ray; ECIRS, endoscopic combined intrarenal surgery; PCNL, percutaneous nephrolithotomy; US, ultrasound; CT, computed tomography; SFR, stone-free rate**

**Supplementary Table 2. SFR definitions**
